# Dynamic changes in clinical and CT characteristics of COVID-19 cases with different exposure histories: a retrospective study

**DOI:** 10.1186/s12879-020-05306-x

**Published:** 2020-08-03

**Authors:** Ruili Li, Guangxue Liu, Xiaojie Huang, Cuiyu Jia, Zhenying Xia, Wenyan Song, Xueqin Li, Xing Wang, Hongjun Li

**Affiliations:** 1 Department of Radiology, Beijing YouAn Hospital, Capital Medical University, No.8 Xi Tou Tiao Youanmen Wai, Fengtai District, Beijing, 100069 China; 2 Department of Natural Medicines, School of Pharmaceutical Sciences, Peking University Health Science Center, No. 38 Xueyuan Road, Haidian District, Beijing, 100191 China; 3 Center for Infectious Diseases, Beijing YouAn Hospital, Capital Medical University, No.8 Xi Tou Tiao Youanmen Wai, Fengtai District, Beijing, 100069 China

**Keywords:** COVID-19, Different exposure histories, CT, Clinical characteristic, Dynamic change

## Abstract

**Background:**

To assess the dynamic changes in clinical and CT characteristics of COVID-19 patients with different epidemiology histories.

**Methods:**

Fifty-three discharged COVID-19 patients were enrolled at Beijing YouAn Hospital, Capital Medical University, between January 21 and March 10, 2020. Spearman correlation analysis was performed between CT scores and laboratory indicators. Patients were divided into the Wuhan group (lived in or with travel to Wuhan, numbering 30 cases) and non-Wuhan group (close contacts or unknown exposure, totaling 23 cases). The CT and laboratory findings were compared between and within groups during the clinical process.

**Results:**

Fever (88.7%), cough (64.2%), fatigue (34%), and abnormal laboratory indicators, including lymphopenia, reduced albumin, albumin/globulin (A/G), and elevated C-reactive protein (CRP), were mainly observed. Subpleural ground-glass opacities (86.8%) were usually detected at admission. The CT scores were highly correlated with lymphocytes, CRP, albumin, and A/G at initial and follow-ups (all *p* < 0.05). Four days after admission, most patients (66.7% Wuhan, 47.8% non-Wuhan) showed progression, and the CT scores of Wuhan significantly increased (*p* = 0.015). Eight days after admission, the vast majority of patients (69.2% Wuhan, 100% non-Wuhan*, p* = 0.006) presented improvement, and the CT scores of non-Wuhan were significantly lower than Wuhan (*p* = 0.006). Pneumonia was completely absorbed in most patients 2–4 weeks after discharge.

**Conclusions:**

CT plays a crucial role in the early diagnosis and monitoring of changes in COVID-19. Lymphocytes, CRP, albumin, and A/G are expected to predict disease severity and prognosis. Viral pathogenicity in non-endemic areas may be weaker than core-infected areas. In most patients, lung lesions can disappear around 4 weeks after discharge.

## Background

In December 2019, an outbreak of novel viral pneumonia occurred in Wuhan, China, which proved to be associated with severe acute respiratory syndrome-related coronavirus 2 (SARS-CoV-2) [1] and was later named by the World Health Organization (WHO) as the corona virus disease (COVID-19). COVID-19 initially mainly affected those who worked or lived around the Huanan seafood market (Wuhan, China) [2, 3]. The number of infected individuals subsequently soared and rapidly spread across the whole country. According to statistics from the National Health Commission of the People’s Republic of China (NHC), up until 10 March 2020, the number of confirmed cases had reached 80,754 [4]. Patients included mainly the residents and the traveler from the affected areas, close contacts, and medical personnel, and the elderly as well as those who had a higher sequential organ failure assessment (SOFA) score, and higher level of d-dimer are the potential risk factors [5]. At the same time, COVID-19 cases were reported and continuously increased in foreign countries. Italy, South Korean, and Iran are the next potential areas for the outbreak [4, 6].

Epidemiological history, clinical manifestations, laboratory tests, chest CT and reverse transcription-polymerase chain reaction (RT-PCR) assay are the major diagnostic components according to NHC guidance [7]. The CT scan also plays a key role in the evaluation of the disease progression. Several cases with the CT features of COVID-19 had been reported [8–13]. Chest CT of most COVID-19 cases (> 70%) shows ground glass opacities with consolidation and interstitial and/or interlobular septal thickening. Recently, the clinical, laboratory and chest CT characteristics between severe and non-severe COVID-19 patients had been compared [14, 15]. However, the information on the dynamic changes in relationships between laboratory data and imaging remains limited. Discussion and analysis of these relationships will ultimately benefit the diagnosis, treatment, and monitoring of COVID-19. Additionally, several studies have indicated that patients outside of Wuhan had a milder infection, compared to published data from Wuhan, the epicenter in China [16–18]. In clinical practice, we have also found similar results that patients who are residents of Wuhan or have recent a travel history to Wuhan, usually showed more prominent CT abnormalities, and progressed in short-term follow-up. However, Lian et al. drew a different conclusion that there was no significant difference between COVID-19 patients with or without Wuhan exposure history in clinical characteristics [19]. The clinical and CT characteristics and their dynamic evolution of patients from non-endemic or core-infected areas have not been systematically reported and are still unclear.

The purpose of this study was to analyze the dynamic change in relationships between laboratory findings and chest CT manifestation of patients with COVID-19, and to explore the clinical, imaging characteristics and outcomes in patients with different epidemiology.

## Methods

### Patients

**Fig. 1:**
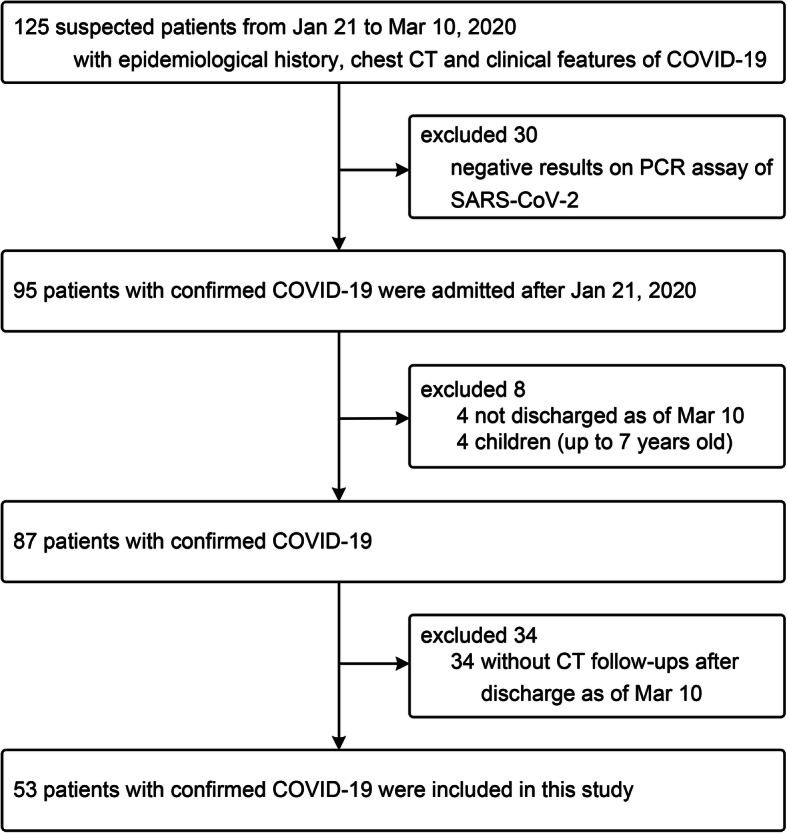
Flow diagram of this study. COVID-19 = Corona Virus Disease, SARS-CoV-2 = severe acute respiratory syndrome-related coronavirus

The ethics committees of Beijing YouAn Hospital, Capital Medical University, approved this retrospective study, which involved no risk for the subjects. Written informed consent was waived. All COVID-19 patients were enrolled from Beijing YouAn Hospital, Capital Medical University, which is one of the designated hospitals for treating COVID-19 patients in Beijing. Inclusion criteria included: (a) confirmation by RT-PCR assay for SARS-CoV-2, which was performed before admission at the Center for Disease Control (CDC), Beijing, China; (b) at least one CT follow-up during hospitalization; (c) at least one CT follow-up after discharge as of March 10, 2020. Exclusion criteria included: (a) influenza A (HIN1, H7N9) and other common bacterial or viral pneumonia; (b) age < 14 years old. A total of 53 patients were included from January 21, 2020 to March 10, 2020. The study flow diagram is presented in Fig. 1. Patients were further divided into 2 groups based on different epidemiological history (Wuhan group: recently lived in or traveled to Wuhan, totaling 30 cases; non-Wuhan group: close contacts or unknown exposure, totaling 23 cases).

### Clinical data collection

Epidemiological, clinical, and laboratory data were obtained from electronic medical records. Detailed clinical information included demographics, current medical history, epidemiological history, past history, symptoms and signs, treatment measures, days of admission. The disease onset date was defined as the day when the symptoms were first noticed. Treatment measures for non-severe patients included symptomatic supportive care (ibuprofen, *lianhua qingwen* capsule, liver protection treatment, correct hypoalbuminemia), antiviral therapy (lopinavir/ritonavir). The *lianhua qingwen* capsule is a Traditional Chinese Medicine (TCM) formula, which has antiviral and anti-inflammatory effects and is used to treat respiratory tract infectious diseases in China [20]. Treatment measures for severe patients included symptomatic supportive care, antiviral therapy, oxygen support, respiratory support, antibiotic therapy, and corticosteroid therapy. Laboratory tests included white blood cell (WBC), lymphocytes (LYM), percentage of LYM (%LYM), neutrophil (NEUT), percentage of NEUT (%NEUT), C-reactive proteins (CRP), procalcitonin (PCT), alanine aminotransferase (ALT), aspartate aminotransferase (AST), albumin (ALB), globulin (GLOB), albumin/globulin ratio (A/G), glomerular filtration rate (eGFR), creatine kinase (CK), influenza antibody tests, and RT-PCR assay for SARS-CoV-2.

### CT image acquisition

The initial CT examinations of all patients were obtained on the day of admission. The median duration from disease onset to CT scan of Wuhan and non-Wuhan cases was 4.5 days (IQR, 3.8–6.3) and 5 days (IQR, 4.0–7.0) respectively. All chest CT scans were performed with a 256-section scanner (Brilliance iCT, Philips Healthcare, Cleveland, OH, USA) without the use of intravenous contrast. The CT examination parameters were as follows: 120 kV; automatic tube current (100 mA–400 mA); iterative reconstruction technique; detector collimation, 128 × 0.625 mm; section thickness, 5 mm; rotation time, 0.4 s; pitch, 0.914; matrix, 512 × 512; and holding breath during end-inspiration. Before January 27, 2020, the chest CT scans were obtained with a slice thickness of 5 mm. To obtain thin-section high resolution images, the chest CT images after January 27, 2020 were reconstructed with a 1.0 mm slice thickness and a 1.0 mm interval. The thin-section multiplanar reconstruction post-processing images in sagittal and coronal positions were then obtained. Two windows were used: lung window (window width, 1500 HU; window level, − 500 HU) and mediastinal window (window width, 350 HU; window level, 50 HU). All follow-up images were obtained through application of the the same protocol as that of the initial scans. The median duration from initial CT to the first follow-up CT scan was 4 days (IQR, 4.0–5.0), and all patients, including the 30 cases in the Wuhan group and the 23 cases in the non-Wuhan group underwent the first follow-up. The median duration from initial CT to the second follow-up CT scan was 8 days (IQR, 7.7–10.0), and 46 patients (26 Wuhan, 20 non-Wuhan) underwent a second follow-up. Seven patients with mild CT abnormalities were discharged without the second follow-up because the results of the first follow-up showed that the lesions were absorbed obviously. As of 10 March 2020, 45 patients (25 Wuhan cases and 20 non-Wuhan cases) had been followed up for approximately 2 weeks after discharge, and 18 patients (12 Wuhan and 6 non-Wuhan) had been followed up for approximately 4 weeks. Ten of these patients (7 Wuhan and 3 non-Wuhan) were followed up at both 2 and 4 weeks after discharge.

### Review of CT images

In the condition of blindness to patients’ clinical information, all CT images were reviewed independently by three radiologists (R.L., C.J., and H.L.) who have between approximately 9 to 32 years of experience in thoracic CT imaging. A final decision was based on consensus when there was a discrepancy in opinion.

The CT images were evaluated for the following features: (1) the presence of pure ground-glass opacification (GGO) (defined as slightly increased lung attenuation with no obscuration of the underlying vascular architecture), patch shadowing (a state between GGO and consolidation, described as GGO density increased with interlobular septal thickening or intralobular networks in GGO, or GGO with consolidation) or consolidation (defined as markedly increased lung attenuation obscuring the underlying vessels); (2) the subpleural or non-subpleural location; (3) the extent of lung involvement, which was assessed independently for each of the five lobes. Each lung lobe was evaluated and assigned a score based on the following: 0 when no involvement, 1 when less than 25% involvement, 2 when 26%—50% involvement; 3 when 51%—75% involvement, and 4 when 76% or more involvement. The total score was the summation of each lobe score (the maximum score being 20), which provided the extent of overall lung involvement. Similar evaluation methods have been reported [9]. The CT scores of lung involvement were also obtained in the follow-ups during hospitalization. The progression or improvement of the disease over time was assessed based on the extent and the density change of lung opacities on CT images.

### Statistical analysis

The Shapiro-Wilk (S-W) test was used to assess the normality of continuous variables. Normally distributed data were presented as means (standard deviation, SD) and were compared between different groups with two independent *t*-test samples. Non-normally distributed data were presented as median (interquartile ranges, IQR) and were compared with the Mann-Whitney U test. Paired *t*-test or two related samples Wilcoxon signed ranks test was used to evaluate the differences of variables between the initial and follow-ups examination. Categorical variables were described as frequency rates or percentages and were compared using χ^2^ or Fisher’s exact test between groups, where appropriate. Spearman correlation was performed for continuous and categorical variables. Two-sided *p* values less than 0.05 were considered statistically significant. All analyses were performed with the use of IBM SPSS Statistics version 20.0 (IBM, Armonk, NY, USA).

## Results

### Demographic and clinical characteristics

The demographic and clinical characteristics are shown in Table 1. The 53 patients included 31 (58.5%) women and 22 (41.5%) men, and the mean age was 50.2 years old (SD, 15.2). The most common complaints were fever (88.7%), cough (64.2%) and fatigue (34%). The common coexisting disorder was hypertension (17%). The median time from onset to admission was 5.0 days (IQR, 4.0–7.0). 76.7% of patients in the Wuhan group were non-severe, while 91.3% of patients in the non-Wuhan group were non-severe. There was no significant difference between the Wuhan and non-Wuhan groups in demographic and clinical characteristics.

**Table 1:** Clinical characteristics of patients with COVID-19 at admission

	All patients (*n* = 53)	^Wuhan (*n* = 30)	^Non-Wuhan (*n* = 23)	*p* value
Age: Mean (SD), years	50.2 (15.2)	52.2 (15.1)	47.6 (15.3)	0.279^‡^
Sex, Female: No., (%)	31/53 (58.5%)	16/30 (53.3%)	15/23 (65.2%)	0.384^||^
**Signs and symptoms**: No., (%)				
Fever	47/53 (88.7%)	27/30 (90%)	20/23 (87%)	1.000^||^
< 37.5	4/53 (7.5%)	2/30 (6.7%)	2/23 (8.7%)	1.000^||^
37.5–38.5	30/53 (56.6%)	17/30 (56.6%)	13/23 (56.5%)	0.992^||^
> 38.5				
Cough and sputum production				
Myalgia or arthralgia	10/53 (18.9%)	5/30 (16.7%)	5/23 (21.7%)	0.640^||^
Chill	7/53 (13.2%)	6/30 (20.0%)	1/23 (4.3%)	0.208^||^
Shortness of breath	9/53 (17.0%)	5/30 (16.7%)	4/23 (17.4%)	1.000^||^
Fatigue	18/53 (34.0%)	10/30 (33.3%)	8/23 (34.8%)	0.912^||^
Nasal congestion	6/53 (11.3%)	3/30 (10.0%)	3/23 (13.0%)	1.000^||^
Headache	8/53 (15.1%)	6/30 (20.0%)	2/23 (8.7%)	0.452^||^
Throat discomfort	9/53 (17.0%)	7/30 (23.3%)	2/23 (8.7%)	0.299^||^
Nausea or vomiting	7/53 (13.2%)	4/30 (13.3%)	3/23 (13.0%)	1.000^||^
Diarrhea	1/53 (1.9%)	1/30 (3.3%)	0/23	1.000^||^
**Coexisting disorders**: No., (%)				
Diabetes	4/53 (7.5%)	2/30 (6.7%)	2/23 (8.7%)	1.000^||^
Hypertension	9/53 (17.0%)	4/30 (13.3%)	5/23 (21.8%)	0.661^||^
Cardiovascular disease	3/53 (5.7%)	2/30 (6.7%)	1/23 (4.3%)	1.000^||^
Hyperlipidemia	1/53 (1.9%)	0/30	1/23 (4.3%)	0.434^||^
Hepatitis B infection	3/53 (5.7%)	3/30 (10.0%)	0/23	0.249^||^
Cancer	3/53 (5.7%)	2/30 (6.7%)	1/23 (4.3%)	1.000^||^
**Time from onset to admission**, Median (IQR)	5.0 (4.0–7.0)	4.5 (3.8–6.3)	5.0 (4.0–7.0)	0.364^‡‡^
**^Disease severity**: No., (%)				
Non-severe	44/53 (83.0%)	23/30 (76.7%)	21/23 (91.3%)	0.299^||^
Severe	9/53 (17.0%)	7/30 (23.3%)	2/23 (8.7%)	

Note: *IQR* Interquartile range

^^^ Wuhan: patients who lived in/or travelled to Wuhan recently

^^^ Non-Wuhan: patients who had contact with a confirmed case or unknown exposure

^^^ Disease severity: based on the Guidelines for the Diagnosis and Treatment of COVID-19 patients by National Health Commission of the People’s Republic of China [7]

^||^ The differences between rates were tested by χ^2^ or Fisher exact tests, if appropriate

^‡^ Independent samples *t*-test

^‡‡^ Mann-Whitney U test

Significant level *p* < 0.05

### CT and laboratory findings at admission

**Fig. 2:**
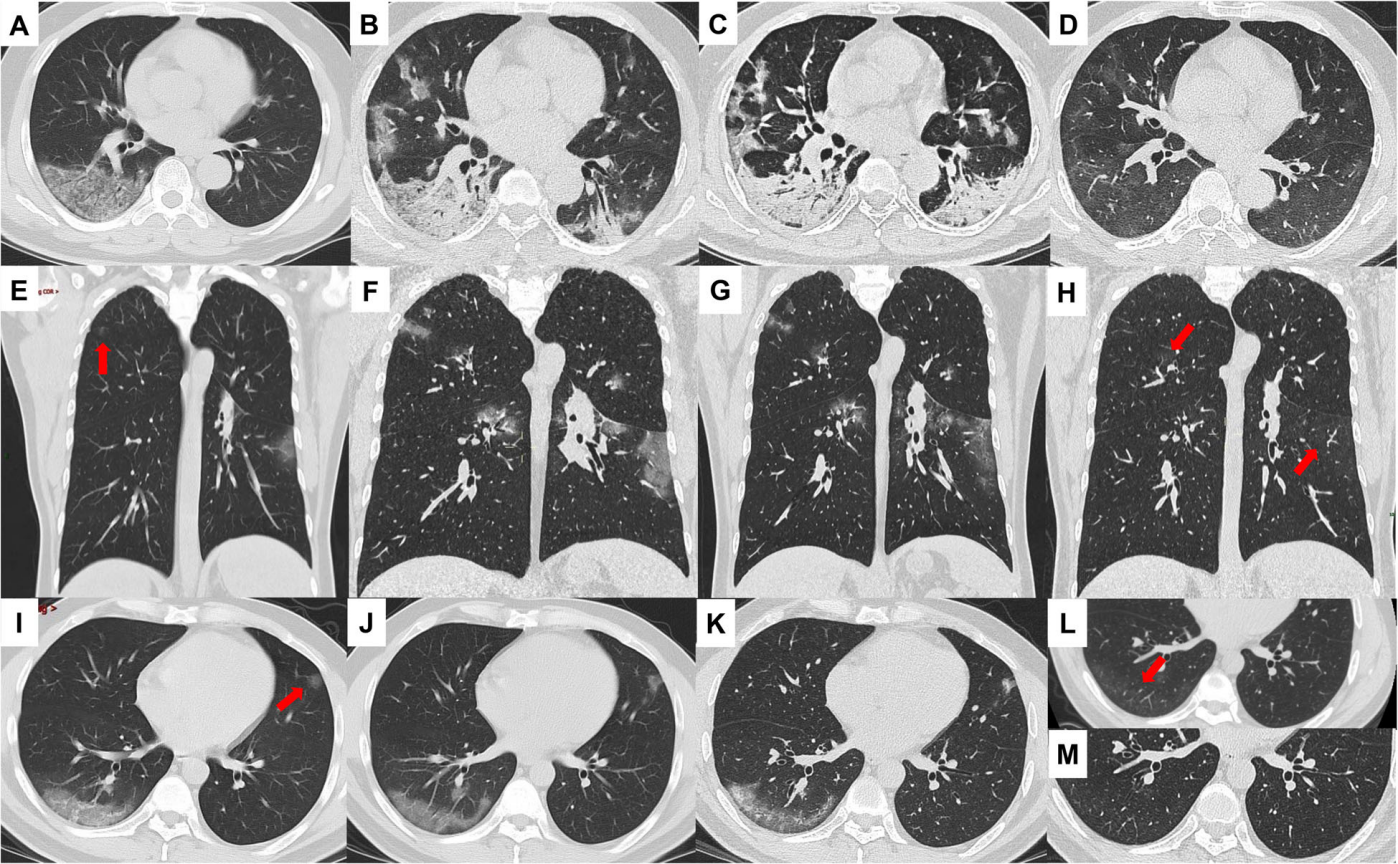
CT images of COVID-19 patients with different exposure history. Axial CT images of a patient living in Wuhan, Hubei, who arrived in Beijing 9 days before admission (**a-d**). The initial CT image showed ground-glass opacity and patchy shadowing, with air bronchial signs, in the right lower lobe (day 6 after symptom onset) (**a**). Image obtained 4 days after admission showed the increased size, number and density of the lesions in both lungs (day 10 after symptom onset) (**b**). Follow-up CT image 8 days after admission showed progressive consolidation (day 14 after symptom onset) (**c**). Follow-up CT image 18 days after discharge showed obvious absorption with ground-glass opacity (day 45 after symptom onset) (**d**). Coronal CT images of a patient with a short travel history to Wuhan, Hubei, 4 days before admission (**e-h**). The initial CT image exhibited ground-glass opacity in the left lower lobe and right upper lobe (red arrow) (day 3 after symptom onset) (**e**). CT images 4 days after admission exhibited that the size, number and density of the lesions increased (day 7 after symptom onset) (**f**). CT images 8 days after admission exhibited that the density of the lesions decreased (day 11 after symptom onset) (**g**). CT images 24 days after discharge exhibited almost complete absorption with only light ground-glass opacity in the left lower lobe and right upper lobe (red arrows) (day 36 after symptom onset) (**h**). Axial CT images of a patient, who had not visited Wuhan, but did have contact with a confirmed patient from Wuhan, 10 days before admission (**i-m**). CT image at admission presented as ground-glass opacity with strip-shaped consolidation in the right lower lobe and ground-glass opacity in the tongue segment of the left superior lobe (red arrow) (day 6 after symptom onset) (**i**). Follow-up CT image 4 days after admission showed decreased lesion density (day 10 after symptom onset) (**j**). CT image on day 8 day after admission presented as slight absorption with strip shadowing (day 14 after symptom onset) (**k**). Follow-up CT image 16 days after discharge showed almost complete absorption with only light ground-glass opacity (red arrow) (day 31 after symptom onset) (**l**). Follow-up CT image 30 days after discharge showed complete absorption (day 45 after symptom onset) (**m**). Notes: The CT images **a**, **e**, **i** and **j** were provided with slice thickness of 5 mm, the rest CT images were thin-section high resolution

Data for CT and laboratory findings at admission are listed in Table 2. Ground-glass opacity (86.8%) and patchy shadowing (50.9%) were the main findings, and the most common location (88.7%) was subpleural (Fig. 2). The median CT score of lung involvement was 4 (IQR, 3.0–6.0). Most patients presented elevated levels of CRP (13.7, IQR 4.1–30.0), decreased LYM (1.13, SD 0.49), ALB (36.7, SD 3.7) and A/G ratio (1.02, SD 0.20). Compared to the non-Wuhan group, while the CRP and CT score of lung involvement in the Wuhan group had a tendency to increase, and the lymphocytes, albumin, and A/G had a tendency to decrease, there was no statistically significant difference.

**Table 2:** CT and laboratory characteristics of patients with COVID-19 at admission

	All patients (n = 53)	^Wuhan (n = 30)	^Non-Wuhan (n = 23)	*p* value
**Chest CT**: No., (%)				
Ground-glass opacity	46/53 (86.8%)	28/30 (93.3%)	18/23 (78.3%)	0.231^||^
Patchy shadowing	27/53 (50.9%)	14/30 (46.7%)	13/23 (56.5%)	0.477^||^
Consolidation	7/53 (13.2%)	3/30 (10%)	4/23 (17.4%)	0.705^||^
Subpleural	47/53 (88.7%)	26/30 (86.7%)	21/23 (91.3%)	0.928^||^
**CT score of lung involvement**				
Median (IQR)	4.0 (3.0–6.0)	4.0 (3.8–6.0)	3.00 (2.0–5.0)	0.322^‡‡^
**Laboratory findings** Mean (SD) or Median (IQR)				
White blood cell, NR: 3.5–9.5 × 10^9^/L	4.01 (3.46–4.76)	3.98 (3.17–5.05)	4.02 (3.54–4.65)	0.753^‡‡^
Lymphocyte, NR: 1.1–3.2 × 10^9^/L	1.13 (0.49)	1.08 (0.48)	1.19 (0.51)	0.418^‡^
Lymphocyte percent, NR: 20–50%	28.4 (12.3)	27.9 (12.2)	29.1 (12.7)	0.731^‡^
Neutrophil, NR: 1.8–6.3 × 10^9^/L	2.30 (1.79–2.98)	2.15 (1.75–2.93)	2.42 (2.02–3.11)	0.490^‡‡^
Neutrophil percent, NR: 40–75%	60.5 (13.1)	60.5 (13.1)	60.5 (13.5)	0.991^‡^
C-reactive protein, NR < 3 mg/L	13.7 (4.1–30.0)	15.0 (8.4–24.8)	13.7 (2.0–51.1)	0.809^‡‡^
Procalcitonin, NR < 0.1 ng/mL	0.11 (0.10–0.12)	0.11 (0.10–0.12)	0.11 (0.10–0.12)	0.372^‡‡^
Alanine aminotransferase, NR: 9–50 U/L	28.0 (20.0–49.0)	28.5 (19.8–45.5)	28.0 (23.0–51.0)	0.548^‡‡^
Aspartate aminotransferase, NR: 15–40 U/L	28.0 (22.0–39.0)	28.0 (22.0–35.0)	29.0 (19.0–42.0)	0.986^‡‡^
Albumin, NR: 40-55 g/L	36.7 (3.7)	36.2 (3.2)	37.3 (4.3)	0.304^‡^
Globulin, NR: 20-40 g/L	36.4 (33.0–40.0)	36.3 (33.0–41.4)	36.5 (33.0–38.3)	0.554^‡‡^
Albumin / globulin ratio, NR: 1.2–2.4	1.02 (0.20)	0.99 (0.19)	1.06 (0.21)	0.226^‡^
Glomerular filtration rate, NR > 90 ml/min/1.73m^2^	104.3 (93.7–112.9)	100.1 (92.5–109.0)	108.6 (93.6–117.1)	0.170^‡‡^
Creatine kinase, NR: 50–310 U/L	61.0 (42.0–116.0)	59.0 (39.0–99.5)	70.0 (45.0–134.0)	0.206^‡‡^

Note: *NR* Normal range

^^^ Wuhan: patients who lived in/or travelled to Wuhan recently

^^^ Non-Wuhan: patients who had contact with a confirmed case or unknown exposure

^||^ The differences between rates were tested by χ^2^ or Fisher exact tests, if appropriate

^‡^ Independent samples *t*-test

^‡‡^ Mann-Whitney U test

Significant level *p* < 0.05

### Changes in CT manifestation and laboratory parameters during hospitalization

It was noticeable that the disease improvement or progression ratio of the Wuhan and non-Wuhan groups were different, based on the extent and density change of lung opacities on chest CT images. In total 66.7% of patients (20/30) in the Wuhan group and 47.8% of patients (11/23) in the non-Wuhan group showed progression at the first follow-up of 4 days (Table 3, Fig. 2). The improvement ratio of the non-Wuhan group (100%, 20/20) was higher than that of the Wuhan group (69.2%, 18/26) on the CT follow-up of 8 days (*p* = 0.006) (Table 3, Fig. 2).

**Table 3:** Follow-ups during hospitalization and after discharge of patients with COVID-19

	All patients	^Wuhan	^Non-Wuhan	*p* value
^**^**^**Follow-ups during hospitalization**: No., (%)				
Follow up 1 (4 days after admission)				
Progression	31/53 (58.5%)	20/30 (66.7%)	11/23 (47.8%)	0.168^||^
Improvement	22/53 (41.5%)	10/30 (33.3%)	12/23 (52.2%)	
Follow up 2 (8 days after admission)				
Progression	8/46 (17.4%)	8/26 (30.8%)	0/20	0.006^||*^
Improvement	38/46 (82.6%)	18/26 (69.2%)	20/20 (100%)	
**Mean length of stay hospital**: Mean (SD)				
	13.9 (4.1)	14.5 (4.7)	13.2 (3.2)	0.213^‡^
**Median interval between symptom onset and discharge**: Median (IQR)				
	19.0 (16.0–23.0)	20.0 (15.8–23.3)	18.0 (16.0–20.0)	0.171^‡‡^
^**^**^**Follow-ups after discharge**: No., (%)				
Follow up 1 (2 weeks after discharge)				
Partly absorption of lesions	41/45 (91.1%)	25/25 (100%)	16/20 (80%)	0.033^||*^
Complete absorption of lesions	4/45 (8.9%)	0/25	4/20 (20%)	
Follow up 2 (4 weeks after discharge)				
Partly absorption of lesions	4/18 (22.2%)	3/12 (25%)	1/6 (16.7%)	1.000^||^
Complete absorption of lesions	14/18 (77.8%)	9/12 (75%)	5/6 (83.3%)	

Note:

*IQR* Interquartile range

^^^ Wuhan: patients who lived in/or travelled to Wuhan recently

^^^ Non-Wuhan: patients who had contact with a confirmed case or unknown exposure

^^^ Follow-ups: based on the extent and density change of lung opacities of chest CT images

^||^ The differences between rates were tested by χ^2^ or Fisher exact tests, if appropriate

^‡^ Independent samples *t*-test

^‡‡^ Mann-Whitney U test

* Significant level *p* < 0.05

**Fig. 3:**
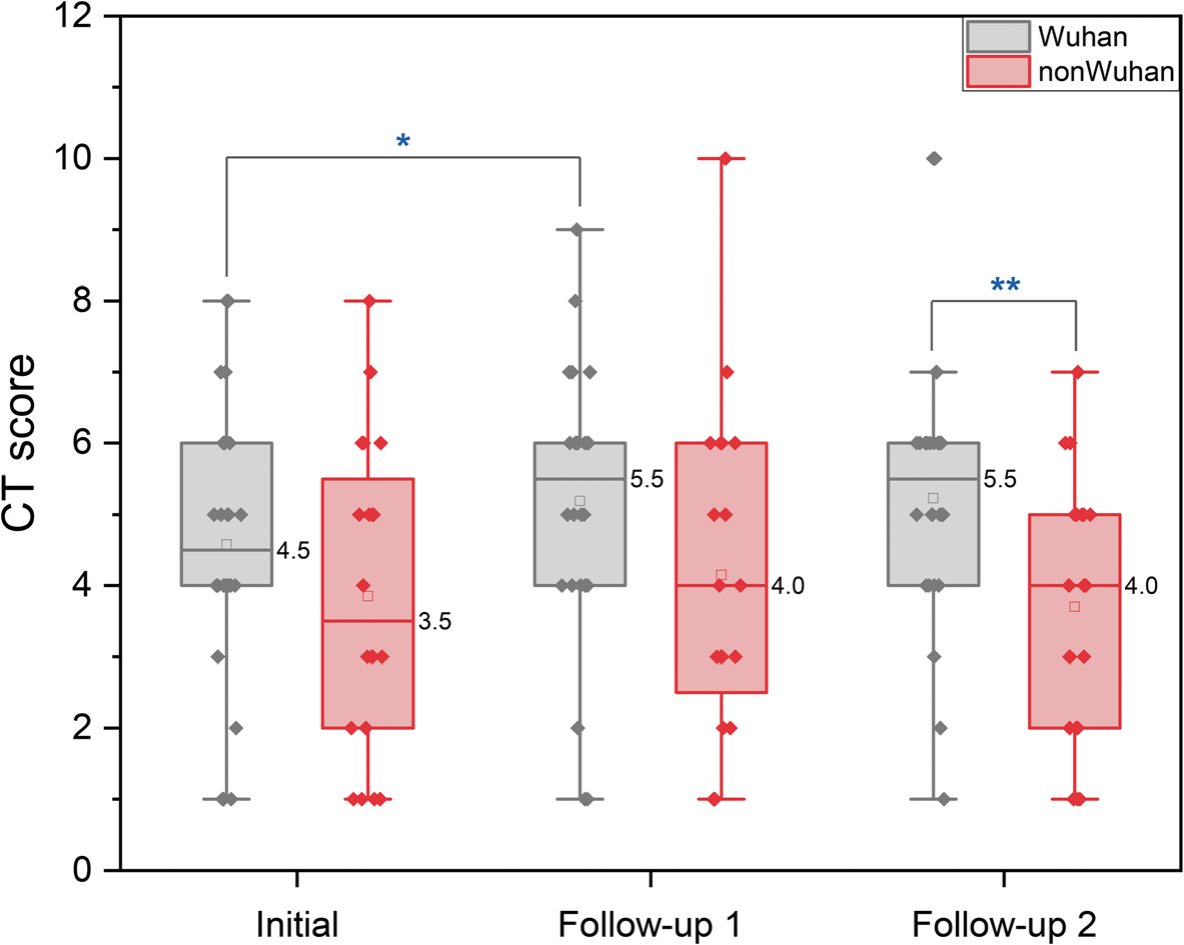
Dynamic changes in lung involvement CT scores in 46 COVID-19 patients during hospitalization. A total of 46 patients (26 Wuhan, 20 non-Wuhan) had completed CT scans at admission, first follow-up of on day 4, and the second follow-up on day 8. The CT scores were compared between and within groups during clinical process. Statistics in Box Plot was presented as median. *, *P* < 0.05; **, *P* < 0.01. Group “Wuhan” indicates patients who lived in or traveled to Wuhan recently. Group “non-Wuhan” indicates patients who had contact with confirmed case or unknown exposure

To further evaluate the dynamic changes in CT characteristics during the disease process, we analyzed the imaging findings from 46 patients (26 Wuhan cases and 20 non-Wuhan cases), who had undergone all chest CT examinations, including admission, follow-up of days 4, and second follow-up of days 8. The within-group comparison showed that the CT scores of lung involvement in Wuhan group increased at the first follow-up (*p* = 0.015) compared to the examination at admission (Fig. 3, Table S1), which suggested disease progression in the short-term follow-up. Comparison of the Wuhan and non-Wuhan groups showed that the CT scores of the non-Wuhan group were significantly lower than that of the Wuhan group at the second follow-up (*p* = 0.006) (Fig. 3, Table S2).

**Fig. 4:**
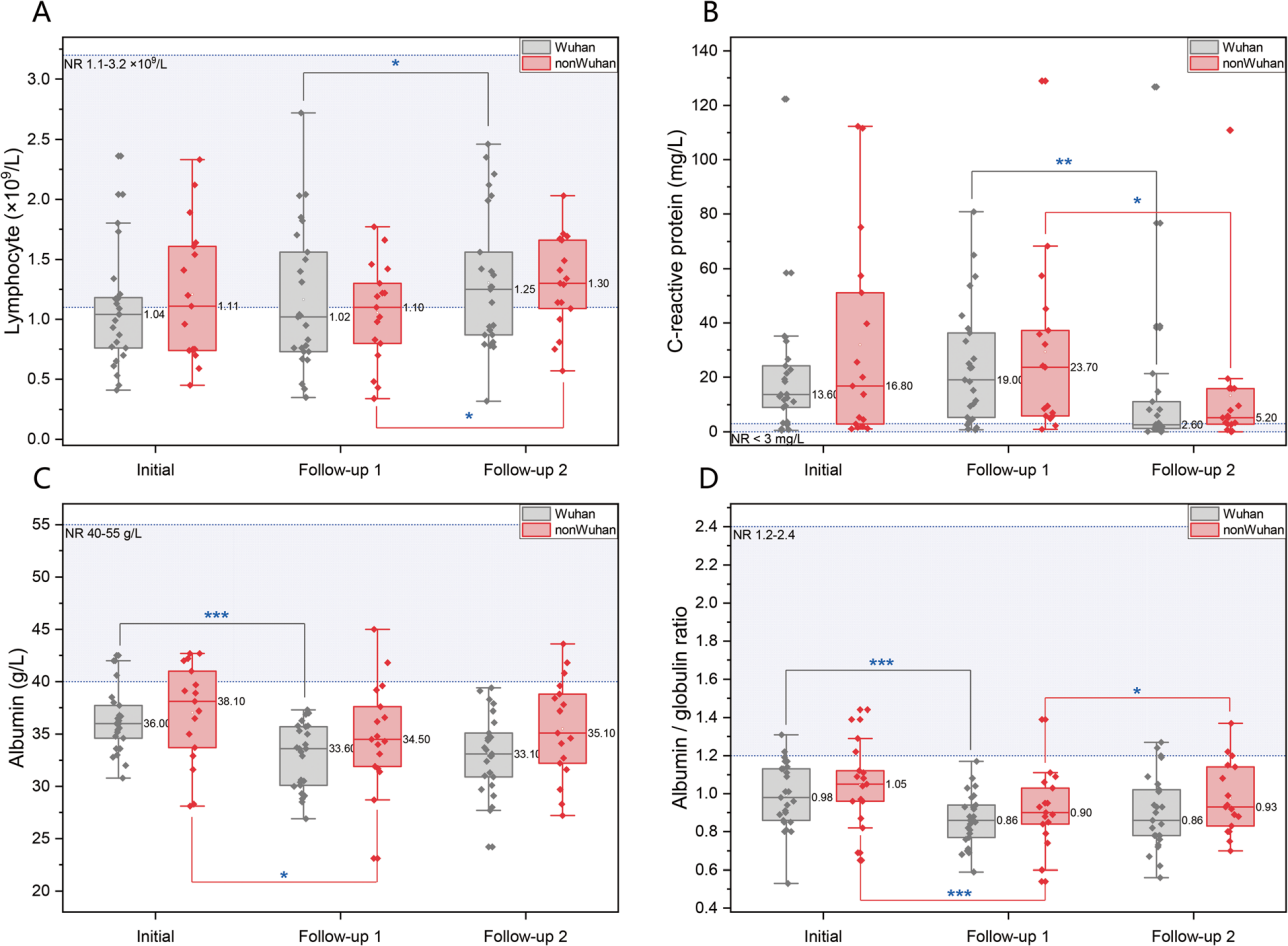
Dynamic changes in laboratory parameters in 42 COVID-19 patients during hospitalization. A total of 42 patients (25 Wuhan, 17 non-Wuhan) had completed examinations at admission, first follow-up on day 4, and the second follow-up on day 8. The laboratory indicators were analyzed between and within groups during the clinical process (sub-figures **a, b, c, d** represents Lymphocytes, C-reactive protein, Albumin, and Albumin / globulin ratio respectively). Statistics in the Box Plot were presented as median. The dotted lines indicate the upper and lower normal limits for each parameter. *, *P* < 0.05; **, *P* < 0.01; ***, *P* < 0.001. NR, normal range. Group “Wuhan” indicates patients who lived in or traveled to Wuhan recently. Group “non-Wuhan” indicates patients who had contact with confirmed cases or unknown exposure

As with CT features, we also analyzed the laboratory indicators of 42 patients (25 Wuhan cases and 17 non-Wuhan cases), who had undergone all examinations at admission, follow-up at day 4, and a second follow-up at day 8. The level of ALB and A/G of Wuhan group significantly decreased at the first follow-up (*p* < 0.001, *p* < 0.001), and similar results were observed in the non-Wuhan group (*p* = 0.014, *p* < 0.001) (Fig. 4, Table S3). Compared to the first follow-up, the LYM significantly increased (*p* = 0.043), the CRP significantly decreased in the Wuhan group (*p* = 0.005) at the second follow-up. For the non-Wuhan group, LYM and A/G significantly increased (*p* = 0.019 and 0.035), while the CRP significantly decreased (*p* = 0.015) (Fig. 4, Table S3). Changes in the above indicators at the second follow-up show an improvement in the clinical condition. There was no significant difference in laboratory indicators between the Wuhan and non-Wuhan groups at initial examination, or at follow-up on day 4 or 8.

### Correlation analysis between lung involvement CT scores and laboratory findings during hospitalization

**Fig. 5:**
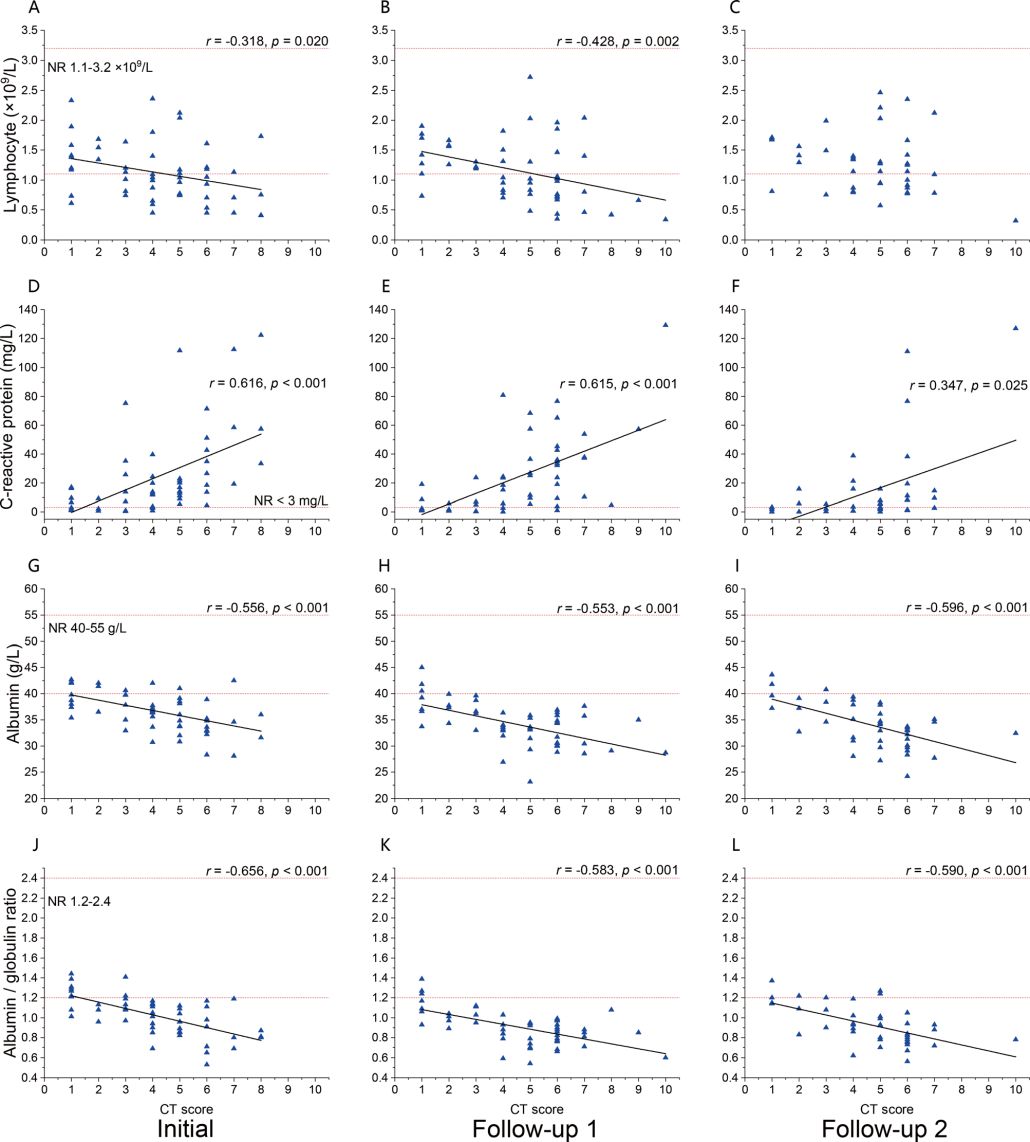
Correlation of lung involvement CT scores and laboratory findings during hospitalization. The CT scores of lung involvement were negatively correlated with lymphocyte (**a, b, c**), albumin (**g, h, i**), albumin / globulin ratio (**j, k, l**), and positively correlated with C-reactive protein (**d, e, f**) at initial examination and follow-ups. Abbreviations: NR, normal range

At initial examination, the CT scores of lung involvement were negatively correlated with LYM (*r* = − 0.318, *p* = 0.020), ALB (*r* = − 0.556, *p* < 0.001), A/G (*r* = − 0.656, *p* < 0.001), and positively correlated with CRP (*r* = 0.616, *p* < 0.001), respectively (Fig. 5). Spearman correlation was performed between the CT score and laboratory finding of all 53 cases.

At the first follow-up of days 4, the CT scores of lung involvement were negatively correlated with LYM (*r* = − 0.428, *p* = 0.002), ALB (*r* = − 0.553, *p* < 0.001), A/G (*r* = − 0.583, *p* < 0.001), and positively correlated with CRP (*r* = 0.615, *p* < 0.001), respectively (Fig. 5). A total of 53 cases underwent the first CT follow-up, but 1 case was dropped due to the absence of laboratory tests at the point of the first CT follow-up. Therefore, the Spearman correlation was performed with the indicators of 52 patients.

The CT scores of lung involvement were negatively correlated with ALB (*r* = − 0.596, *p* < 0.001), A/G (*r* = − 0.590, *p* < 0.001), and positively correlated with CRP (*r* = 0.347 and 0.025) at the second follow-up (8 days later), respectively (Fig. 5). A total of 46 cases underwent the second CT follow-up, 4 cases were dropped due to the absence of laboratory tests. Therefore, the spearman correlation test was performed between the CT scores and laboratory indices of 42 patients.

### Follow-ups after discharge

In this study, the average length of hospital stay was 13.9 days (SD, 4.1), and the median interval between symptom onset and discharge was 19.0 days (IQR, 16.0–23.0). Compared with the non-Wuhan group, the average length of hospital stay and interval between symptom onset and discharge in the Wuhan group had a tendency to be longer but the difference was not statistically significant.

Forty-five patients (25 Wuhan cases and 20 non-Wuhan cases) were first followed up of 2 weeks after discharge. The CT images show partly absorption with decreased size and density of the lesions in all Wuhan cases (100%, 25/25) and 80% non-Wuhan cases (80%, 16/20) (Fig. 2). Moreover, the complete absorption of lesions in 4 non-Wuhan cases (20%, 4/20) is shown in Table 3. Subsequently, 10 of 45 patients (7 Wuhan cases and 3 non-Wuhan cases) were followed up at 4 weeks after discharge. Complete absorption of lesions was shown on the CT imaging of 4 Wuhan patient and 2 non-Wuhan patients (Fig. 2), and CT images of the other 3 Wuhan patients and 1 non-Wuhan patient still exhibited small-scale lesions. Eight patients (5 Wuhan cases and 3 non-Wuhan cases) were followed up only 4 weeks after discharge, and the complete absorption of lesions was found. As with the CT images, laboratory indicators of all patients such as CRP, lymphocyte, albumin and A/G ratio returned to normal between 2 to 4 weeks after discharge, and the RT-PCR assay results of all patients after discharge were negative.

## Discussions

Consistent with former studies and reports, the clinical symptoms of COVID-19 patients in our research were atypical and similar to the common cold or influenza. The onset of symptoms mainly included fever (88.7%), cough (64.2%) and fatigue (34%), and some patients with COVID-19 had lymphopenia [2, 14–16, 21, 22]. In this study, decreases in not only LYM but also ALB and A/G were observed, and the CRP level was elevated at the same time.

Moreover, LYM, ALB and A/G were significantly negatively correlated with CT scores of lung involvement, the CRP level positively were correlated with the CT scores of lung involvement at the initial examination and the follow-ups. Recent research also demonstrated that severe patients had more prominent laboratory abnormalities (i.e., lymphopenia, elevated CRP levels) than non-severe patients [15]. The results indicated that the levels of LYM, ALB, A/G, and CRP were significantly correlated with the severity of the COVID-19. These indicators combined with CT features are expected to benefit the early diagnosis and prognosis of COVID-19. Furthermore, LYM, ALB, A/G and CRP could also be used to assess the progression and predict prognosis.

Ground-glass opacity (86.8%) and patchy shadowing (50.9%) under the pleura were most common during the initial CT scan. One of our findings was that the CT images of most patients (especially in the Wuhan group) showed an increase in lesion density and size at the short-term follow-up (4 days after admission), suggesting disease progression, although all patients received regular treatments. Changes in laboratory indicators (increased CRP, decreased LYM, ALB, A/G) also suggested progression. Recent studies from Wuhan also found that most patients exhibited progression in early stage follow-up [12, 21]. One possible explanation is that the coronaviruses interact with and modify the host intracellular environment during infection for rapid replication. Despite careful treatment, time is necessary for COVID-19 patients to build the immune response and produce antibodies to inhibit viral replication. As a result, it is important to control the progression in the early stages of the disease with the utmost effort.

According to the treatment guidelines of COVID-19, RT-PCR assay is the gold standard for the diagnosis of COVID-19 for all patients [7]. The nasopharyngeal swab had been adopted most widely in clinical practice because of its convenience. Recently, however, it was clinically found that some suspected patients’ throat swabs were SARS-CoV-2 negative after repeated RT-PCR tests, while the clinical symptoms and CT manifestation of them were consistent with the performance of COVID-19. In the end, the RT-PCR showed SARS-CoV-2 positive after repeated assays or test with deep sputum. The false-negative results in the RT-PCR assay of respiratory secretions may be caused by the low viral load of SARS-CoV-2 in testing samples [23–27]. Both the upper and lower respiratory tract specimens should be analyzed to increase the sensitivity of the test. However, sampling from the lower respiratory tract is not easy to obtain.

Due to the limitations of respiratory tract specimens, over-dependence on the results of RT-PCR assay will lead to the delay in diagnosis and treatment to a certain extent, and will affect the control of COVID-19 pandemic eventually. It is particularly important to combine epidemiological history, clinical symptoms, laboratory indicators, and CT findings. A detailed history of exposure is critical for the diagnosis. In the correct clinical and laboratory setting, such as fever, cough, progressive lymphopenia and hypoalbuminemia, negative for the other common respiratory pathogens, a possible diagnosis of COVID-19 should be considered for patients with ground-glass opacities or consolidation on chest CT images.

Age, viral load, lung injury score, and albumin, CRP, LDH, LYM (%), LYM, and NEU (%), may be predictors of disease severity [28], and lymphopenia is linked to the increased severity, mortality and dysregulated immunological response [29, 30]. In our study, there was no significant difference in age, gender or duration from onset to admission between the Wuhan and non-Wuhan groups. However, patients in the Wuhan group had a higher tendency of lung involvement CT scores and a lower tendency of LYM, ALB, and A/G at the initial examination and the follow-ups during hospitalization. Noteworthy is that the proportion of severe patients in the Wuhan group was 23.3%, and most of them showed progression (66.7%) by increasing involvement range and density of lung opacities on CT imaging at the first follow-up at 4 days, and 69.2% of the patients showed improvement at the second follow-up at 8 days. For patients in the non-Wuhan group, the proportion of severe patients was 8.7%, and about half of the patients’ CT imaging (47.8%) showed progression at the first follow-up, but the improvement totaled 100% at the second follow-up.

A single-center study from Wuhan University’s Zhongnan Hospital reported a higher proportion of severe patients (26%) [14]. Xu et al. reported that compared with COVID-19 patients initially infected in Wuhan, the symptoms of patients in other regions were relatively mild [16]. One possible reason is limited medical resources. Wuhan is a high-endemic area, and the number of confirmed cases has dramatically increased so there may be a lack of medical resources. Another possible explanation is that the virulence of SARS-CoV-2 may diminish during transmission. Like the severe acute respiratory syndrome coronavirus (SARS-CoV) and the middle east respiratory syndrome coronavirus (MERS-CoV), due to error-prone RNA-dependent RNA polymerase (RdRP) of coronaviruses, SARS-CoV-2 is also prone to mutation and recombination [31, 32]. We speculate that the adaptive evolution of SARS-CoV-2 has occurred in transmission, resulting in the change of capacity to cause disease. Viral virulence in patients with COVID-19 from Wuhan may be different from that of infected patients who have not been to Wuhan. The viral load in serum or other body liquids might be a useful marker related to disease severity of SARS-CoV-2 infection. Further research is needed to confirm this speculation.

According to the follow-up laboratory indicators and CT manifestations, though most patients presented progression 4 days after admission, most of them exhibited improvement 8 days after admission, indicating that the disease can be controlled with timely treatment. To date, no drugs for the effective treatment of COVID-19 have been approved [33, 34]. Treatment is individualized according to the severity of the condition and individual heterogeneity. Treatment measures mainly include symptomatic supportive care, antiviral therapy (lopinavir/ritonavir), oxygen support, respiratory support, antibacterial drugs, and an appropriate dose of corticosteroid therapy. Traditional Chinese Medicine (TCM) is recommended for symptomatic treatment.

Patients can be discharged if they meet the following conditions: body temperature returned to normal at least for 3 days, significant improvement in respiratory symptoms, chest CT performance improved significantly, and respiratory specimens (sputum and nasopharyngeal) nucleic acid tests were negative for consecutive twice (sampling interval at least 1 day). The average length of hospitalization was 13.9 days and the median interval between symptom onset and discharge was 19 days. According to the diagnosis and treatment guidelines [7], self-isolation for 2 weeks after discharge, and follow-up hospital visits at 2 and 4 weeks after discharge are recommended. The prognosis of patients with COVID-19 at our institution is satisfactory. We found that the lung lesions of most patients were partially absorbed within 2 weeks after discharge. Complete absorption of the lesions was observed 2 weeks after discharge in a few non-Wuhan patients and after 4 weeks for most patients. Moreover, it may take longer to complete the absorption of pneumonia for a couple of severer patients. A study on the prognosis of SARS revealed that pulmonary fibrosis in 62% of patients (15/24) was found at about 5 weeks after discharge [35]. Although the fibrotic change was not found in this study, we have to be cautious about COVID-19, due to the infectivity of some patients with negative results of RT-PCR assay and lung lesions which were not fully absorbed [36]. Therefore, at least 4 weeks are necessary for self-quarantine after discharge as far as possible, which may contribute to the reduction of human-to-human infection.

Our study has several limitations. First, the study subjects were limited to discharged patients who had at least two CT scans during hospitalization and at least 1 CT scan after discharge to ensure more information on clinical and CT features. Thus, the sample size was relatively small and there may be selection biases, further research with larger cohorts is needed to verify our results. Second, our results should be interpreted with caution, because 4 elderly critically ill patients (median age 72.5 years old) still receiving treatment in the respiratory intensive care unit (RICU) and 4 infected children with normal chest CTs were not included. Third, various lung lobes have different volumes, which may have different effects on lung CT score. Therefore, the CT score of lung involvement based on the anatomical basis of the five lobes may not be sufficiently precise. In addition, it is only related to the range of affected areas, the changes in the density of the lesion cannot be quantified and need to be improved in future research. Fourth, the viral load and other laboratory indicators, as the potential markers related to the disease severity of COVID-19, should be assessed. Moreover, the role of adaptive evolution in reducing pathogenicity of SARS-CoV-2 needs to be considered.

## Conclusions

CT is an intuitive method of great value in the early diagnosis and monitoring of changes in COVID-19. Lymphocytes, C-reactive protein, albumin, and albumin/globulin ratio are sensitive indicators of disease progression and prognosis. Viral pathogenicity may differ between non-endemic areas and core infected areas. Although the pneumonia being completely absorbed in most patients 1 month after discharge is satisfactory, continued self-isolation for at least 1 month after discharge as far as possible is necessary.

## Supplementary information

**Additional file 1: Table S1.** Dynamic changes in lung involvement CT scores during hospitalization (*n* = 46). **Table S2.** Comparison of lung involvement CT scores between Wuhan and Non-Wuhan groups at admission, follow-up 1, and follow-up 2 (n = 46). **Table S3.** Dynamic changes in laboratory findings during hospitalization (*n* = 42)

## Data Availability

The material supporting the conclusion of this study has been included in the manuscript. The datasets used and/or analyzed during the current study are available from the corresponding author on reasonable request.

## Abbreviations

SARS-CoV-2: Severe acute respiratory syndrome-related coronavirus 2
COVID-19: Corona virus disease
RT-PCR: Reverse transcription-polymerase chain reaction
WBC: White blood cell
LYM: Lymphocytes
%LYM: Percentage of LYM
NEUT: Neutrophil
%NEUT: Percentage of NEUT
CRP: C-reactive proteins
PCT: Procalcitonin
ALT: Alanine aminotransferase
AST: Aspartate aminotransferase
ALB: Albumin
GLOB: Globulin
A/G: Albumin/globulin ratio
eGFR: Glomerular filtration rate
CK: Creatine kinase
