# Supplementary material for: Dynamic changes in clinical and CT characteristics of COVID-19 cases with different exposure histories: a retrospective study
Source: BMC Infect Dis. 2020 Aug 3;20:567. doi: 10.1186/s12879-020-05306-x (PMC7397456; doi:10.1186/s12879-020-05306-x)
Supplement: Supplementary file 1 — Additional file 1: Table S1. Dynamic changes in lung involvement CT scores during hospitalization (n = 46). Table S2. Comparison of lung involvement CT scores between Wuhan and Non-Wuhan groups at admission, follow-up 1, and follow-up 2 (n = 46). Table S3. Dynamic changes in laboratory findings during hospitalization (n = 42) [file 12879_2020_5306_MOESM1_ESM.docx]

**Supplementary material**

Table S1. Dynamic changes in lung involvement CT scores during hospitalization (n=46)

|  | **Comparison between initial exam and the first follow-up** | | | |  | **Comparison between the two follow-ups** | | | |
| --- | --- | --- | --- | --- | --- | --- | --- | --- | --- |
|  | Initial | Follow-up 1 | *Z* value | *p* value |  | Follow-up 1 | Follow-up 2 | *Z* value | *p* value |
| **CT scores of lung involvement,** Median (IQR) |  |  |  |  |  |  |  |  |  |
| **^Wuhan (n=26)** | 4.5 (4.0-6.0) | 5.5 (4.0-6.0) | -2.434 | 0.015^△*^ |  | 5.5 (4.0-6.0) | 5.5 (4.0-6.0) | -0.119 | 0.905^△^ |
| **^Non-Wuhan (n=20)** | 3.5 (2.0-5.7) | 4.0 (2.2-6.0) | -0.787 | 0.431^△^ |  | 4.0 (2.2-6.0) | 4.0 (2.0-5.0) | -1.930 | 0.054^△^ |

Note:

46 patients with all chest CT at admission, follow-up 1, and follow-up 2 were included.

^ Wuhan: patients lived in/or travelled to Wuhan recently.

^ Non-Wuhan: patients who contacted with confirmed case or unknown exposure.

^△^ Wilcoxon signed ranks test.

**^🞶^** Significant level p<0.05.

Table S2. Comparison of lung involvement CT scores between Wuhan and Non-Wuhan groups at admission, follow-up 1, and follow-up 2 (n=46)

|  | **^Wuhan (n=26)** | **^Non-Wuhan (n=20)** | *p* value |
| --- | --- | --- | --- |
| **CT scores of lung involvement** Median (IQR) |  |  |  |
| Initial | 4.5 (4.0-6.0) | 3.5 (2.0-5.7) | 0.262 |
| Follow-up 1 | 5.5 (4.0-6.0) | 4.0 (2.2-6.0) | 0.069 |
| Follow-up 2 | 5.5 (4.0-6.0) | 4.0 (2.0-5.0) | 0.006^‡‡🞶^ |

Note:

46 patients with all chest CT at admission, follow-up 1, and follow-up 2 were included.

^ Wuhan: patients lived in/or travelled to Wuhan recently.

^ Non-Wuhan: patients who contacted with confirmed case or unknown exposure.

^‡‡^ Mann-Whitney U test.

**^🞶^** Significant level p<0.05.

Table S3. Dynamic changes in laboratory findings during hospitalization (n=42)

|  | **Comparison between initial exam and the first follow-up** | | | |  | **Comparison between the two follow-ups** | | | |
| --- | --- | --- | --- | --- | --- | --- | --- | --- | --- |
|  | Initial | Follow-up 1 | *t* or *Z* value | *p* value |  | Follow-up 1 | Follow-up 2 | *t* or *Z* value | *p* value |
| **^ Wuhan (n=25)** |  |  |  |  |  |  |  |  |  |
| Lymphocyte, NR: 1.1-3.2×10^9^/L | 1.07 (0.49) | 1.16 (0.60) | 1.032 | 0.313^□^ |  | 1.02 (0.73-1.63) | 1.25 (0.84-1.78) | -2.023 | 0.043^△*^ |
| C-reactive protein, NR < 3 mg/L | 13.6 (6.3-25.4) | 19.0 (5.0-37.1) | -1.305 | 0.192^△^ |  | 19.0 (5.0-37.1) | 2.6 (1.3-12.9) | -2.812 | 0.005^△*^ |
| Albumin, NR: 40-55g/L | 36.3 (3.1) | 32.8 (3.1) | -6.512 | <0.001^□*^ |  | 32.8 (3.1) | 33.1 (3.8) | 0.435 | 0.667^□^ |
| Albumin / globulin ratio, NR: 1.2-2.4 | 0.98 (0.86-1.14) | 0.86 (0.77-0.96) | -3.311 | <0.001^△*^ |  | 0.87 (0.14) | 0.90 (0.19) | 1.168 | 0.254^□^ |
| **^ Non-Wuhan (n=17)** |  |  |  |  |  |  |  |  |  |
| Lymphocyte, NR: 1.1-3.2×10^9^/L | 1.05 (0.42) | 1.30 (0.39) | -1.626 | 0.123^□^ |  | 1.05 (0.42) | 1.30 (0.39) | 2.611 | 0.019^□*^ |
| C-reactive protein, NR < 3 mg/L | 31.9 (37.5) | 29.3 (32.8) | -0.370 | 0.717^□^ |  | 23.7 (5.8-41.2) | 5.2 (2.8-15.9) | -2.438 | 0.015^△*^ |
| Albumin, NR: 40-55g/L | 37.0 (4.8) | 34.9 (5.1) | -2.753 | 0.014^□*^ |  | 34.9 (5.1) | 35.5 (4.8) | 0.783 | 0.445^□^ |
| Albumin / globulin ratio, NR: 1.2-2.4 | 1.0 (0.2) | 0.9 (0.2) | -4.919 | <0.001^□*^ |  | 0.91 (0.20) | 0.98 (0.19) | 2.298 | 0.035^□*^ |

Note:

42 patients with all chest CT and laboratory tests at admission, follow-up 1, and follow-up 2 were included.

NR: normal range.

^ Wuhan: patients lived in/or travelled to Wuhan recently.

^ Non-Wuhan: patients who contacted with confirmed case or unknown exposure.

^△^ Wilcoxon signed ranks test was performed when the distribution of paired variables difference was non-normal, and the Shapiro-Wilk test was necessary to verify the normal distribution at first. The variables were shown as mean (SD) when the Paired *t*-test was performed, and median (IQR) when Wilcoxon signed ranks test was used.

^□^ Paired *t*-test was performed when the paired variables difference was normal distribution.

**^🞶^** Significant level p<0.05.
